# Towards solving the missing ice problem and the importance of rigorous model data comparisons

**DOI:** 10.1038/s41467-022-33952-z

**Published:** 2022-10-24

**Authors:** Yusuke Yokoyama, Kurt Lambeck, Patrick De Deckker, Tezer M. Esat, Jody M. Webster, Masao Nakada

**Affiliations:** 1 Atmosphere and Ocean Research Institute, The University of Tokyo, Chiba, 277-8564 Japan; 2 Department of Earth and Planetary Sciences, Graduate School of Science, The University of Tokyo, Tokyo, 113-0033 Japan; 3 Graduate Program on Environmental Sciences, Graduate School of Arts and Sciences, The University of Tokyo, Tokyo, 153-8902 Japan; 4 Biogeochemistry Research Center, Japan Agency for Marine-Earth Science and Technology, Kanagawa, 237-0061 Japan; 5 Research School of Physics, The Australian National University, Canberra, ACT 2601 Australia; 6 Research School of Earth Sciences, The Australian National University, Canberra, ACT 2601 Australia; 7 School of Geosciences, University of Sydney, Sydney, NSW 2006 Australia; 8 Department of Earth and Planetary Sciences, Faculty of Science, Kyushu University, Fukuoka, 819-0395 Japan

**Keywords:** Palaeoceanography, Palaeoclimate

**arising from** E. Gowan et al. *Nature Communications* 10.1038/s41467-021-21469-w (2021)

To reconstruct past sea levels, for estimating changes in global ice volume, it is necessary to obtain evidence from biofacies that rely on in situ fossil material and that grew at specific, shallow-water depths close to the oceans shorelines during their life span. At such sites, a time series is also necessary for estimating the exact timing of the lowest sea level by determining geological sequences and the dating has to be made on the in situ fossil organisms themselves.

The recently published paper by Gowan et al.^1^ (hereafter EJG21) presented a new global ice sheet reconstruction for the last 80,000 years spanning the time period of the Last Glacial Maximum (LGM: around ca. 20 ka) and marine isotope stage 3 (MIS3: 57–30 ka). The reconstructed global ice volume relied on a simple ice model constrained by near-field ice volumes derived from glacio-geological evidence. The two main conclusions arising involved the magnitude of the global mean sea level during the LGM and during MIS 3. Firstly, their LGM sea level at −116 m is significantly shallower than previously reported values (e.g., ref. 2). The authors claim that their new ice volume reconstruction can explain LGM relative sea level (RSL) observations. However, their proposed ice model is inconsistent with other far-field site observations, in particular for two locations in the Bonaparte Gulf (BG) in northwestern Australia^3,4^ and the Great Barrier Reef (GBR)^5^. Secondly, EJG21’s MIS3 sea level is also much shallower than most other records suggest (e.g., ref. 6) and this difference was attributed to a problem with the δ^18^O-based sea-level reconstructions^7^.

We firmly believe that EJG21 have not only misrepresented the earlier results^3–5^ but also their interpretations of previously reported RSLs from far-field sites, such as those from Sunda Shelf, Barbados, GBR and BG, have not been tested against other far-field records such as those from Tahiti and Papua New Guinea^6,8^. In their reduction of the RSL to global mean sea level, EJG21 ignored uncertainties associated with the isostatic adjustment contribution and have not explored the full parameter space of possible ice- and earth-model parameters.

BG is situated at a ‘far-field’ site in terms of distance from former and current glaciated regions and the original study^3^ reported the LGM RSL from the Gulf as being between 120–123 m below present-day sea-level. These depths were derived from rigorous facies analyses using in situ micropalaeontological assemblages. The zoning of the marine organisms’ habitats arises from environmental gradients. The salinity range is often found to be between 0.05 and 3% in coastal brackish environments with numbers of species as well as sizes of some biota being reduced dramatically due to salinity stress associated with maintaining osmotic body pressure of foraminifera and other organisms. The depths between 310–220 cm in the master core GC5 contain both ostracods and benthic foraminifera^3,9^. In particular, the benthic foraminifer *Ammonia* sp. lives in low-salinity, estuarine environments. The micropalaeontological evidence^9^ clearly documents a transition between several facies around the LGM. The sizes of *Ammonia* sp. are abnormally small, reflecting environmental stress caused by low salinity. This was confirmed by the geochemical signatures of sediment cores obtained from BG during a subsequent research cruise in 2011^4^. In particular, radiocarbon dates, shallow-water mollusk analysis (*Anadara* sp., *Paphia undulata*, and others) and geochemical analyses^4^ of the 583 cm core KH11-1GC6 confirmed a local LGM sea level of −120 m (Fig. 1). Even though the site is located in the far-field, it is not immune from GIA^10^. Correcting for GIA results in larger reconstructed LGM ice volumes compared to the estimates by EJG21.

**Fig. 1:**
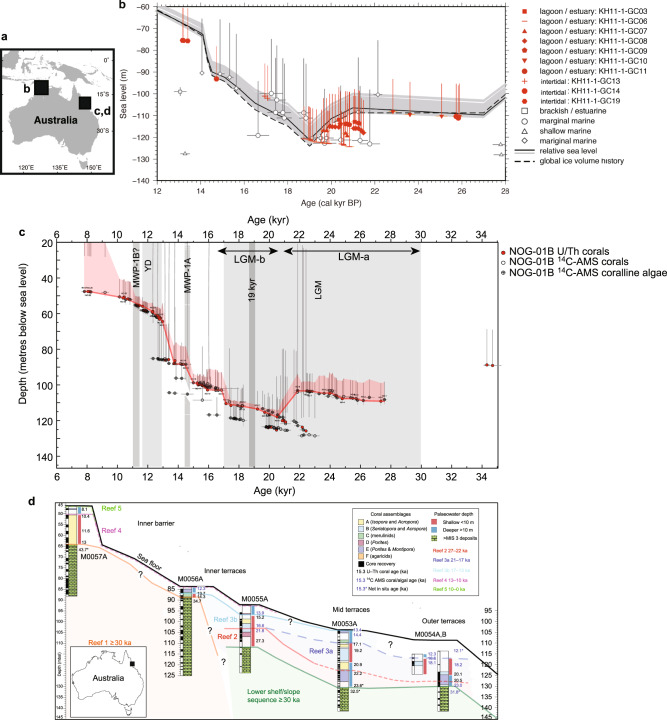
Tightly constrained Last Glacial Maximum (LGM) sea level record from around the Australian coast. **a** Map showing locations of relative sea level (RSL) reconstructions using series of cores obtained from northwestern (Bonaparte Gulf^3,4^) and northeastern Australia (the Great Barrier Reef: GBR^5^). **b** The Bonaparte Gulf cores were retrieved from different water depths designed to capture the full range of LGM RSL. Details of facies analyses were conducted using microfossils and radiocarbon dates that confirmed local RSL to be as low as −120 to −123 m during the LGM. Full reference citation details are available in ref. 4. **c** Corals, foraminifera, and other biofacies analyses, together with >800 radiometric dates, revealed sub-seafloor reef structures and RSL during the last 30,000 years at the GBR^5,11^. Two sigma age uncertainties are shown for each dated sample. RSL curves^5^ for samples drilled at Noggin Pass (NOG) off Cairns (**d**) They are based on extensive palaeo-water depth analyses using habitat depth ranges of various fossils from NOG. Full reference citation details are available in ref. 11.

The depth-transect coring approach used in these studies accurately captured past RSL fluctuations. In particular, since the tidal range during the LGM in the GB was negligible according to high-resolution tidal model simulations^4^. This lead to the conclusion that the lowest LGM RSL at the Gulf is tightly constrained within narrow uncertainties between −120.6 and −124.5 m^4,9^ and clearly deeper than the −116 m attributed by EJG21. By not adding any new evidence and without considering these depositional criteria, EJG21 ignores more than half of the RSL observations available from this locality. In addition, they did not comment on the choice of some of their sea-level indicators which were inconsistent with their own predicted RSL curve.

The offshore underwater fossil GBR reefs were drilled to reconstruct the LGM sea levels in northeastern Australia. Over 800 radiometric dates were obtained to reconstruct the relative sea level spanning the last 30,000 years^5,11^. Depth ranges were conservatively assessed using biofacies analyses supported by both the geomorphology of the seafloor and sub-seafloor reef structures^11^. The GBR LGM RSL at the site off Cairns (NOG) is −118 m (Fig. 1).

As for their BG discussion, EJG21 ignored the uncertainties in sea-level indicators and appear to have adopted a uniform vertical uncertainty estimate that does not reflect the careful and conservative palaeo-water depth estimates from the original publications^5,11^. One of the main conclusions of EJG21 is that GMSL during the MIS3 was higher (implying smaller ice sheets) by 20–80 m than previously reported. Important constraints for the MIS 3 GMSL come from both uplifted and submerged coral reef terraces in Papua New Guinea (PNG), a tectonically-active site, that has been extensively studied with corals from terrace surfaces and drill cores and were mass-spectrometric uranium/thorium dated (e.g., ref. 6.). Distinct reef structures, separated by 10–20 m steps seen in reef sections of different rates of uplift are indicative of sea level and ice sheet fluctuations that, when corrected for tectonic uplift, clearly indicate MIS3 GMSL oscillating between −60 and −90 m below present sea level^6^. Elsewhere, submerged coral reefs in Tahiti drilled by IODP (International Ocean Drilling Program) Expedition 310 also indicate GMSL at −65 to −75 m^12^. These results contradict those tabled by EJG21 (Fig. 2) who quote values of −25 to −60 m based primarily on their simple glacial model and their argument that MIS3 GMSL was significantly shallower than the previous estimations. A better strategy would have been to use these discrepancies to assess the reliability of their ice model and examine what modifications are required to make them consistent with the far-field RSL data.

**Fig. 2:**
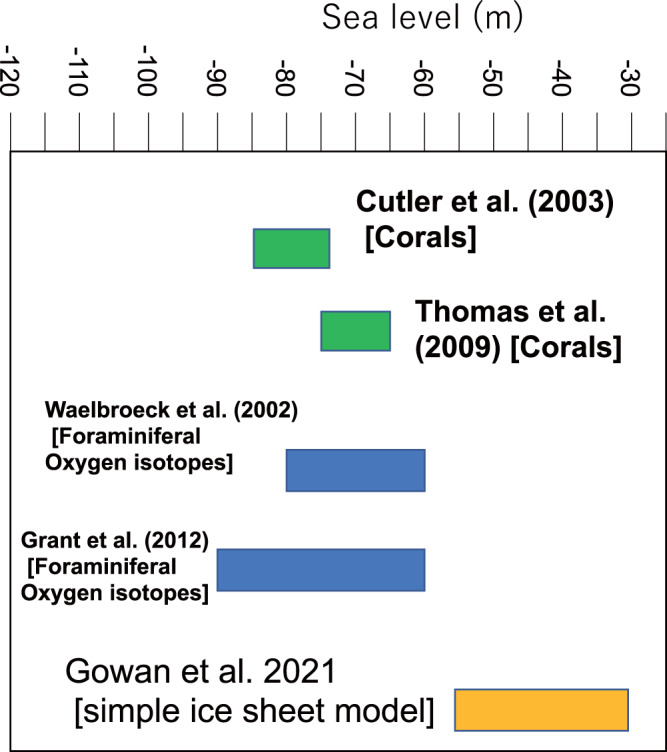
Comparison between recently published MIS3 (Marine Isotope Stage 3) sea level model results, reported by Gowan et al. (EJG21)^1^ and previous actual measurements^6,7,12,15^. There is a clear discrepancy between the two that raises questions about the veracity of the underlying assumptions of the particular model used by EJG 21.

In conclusion, the paper by EJG21 argued that they have solved the missing ice problem of the LGM^13^ based on their ice models and on their invalid re-interpretation of the observational sea-level data. At the same time, EJG21 create a new ice-volume problem for the MIS3 period. In both cases, their smaller ice volume reconstructions, compared to previously published values, are not warranted. Significant progress has been made in ice sheet modeling (e.g., ref. 2), and community efforts have led to better understanding of the GMSL (e.g., PALSEA^14^). Unfortunately, the EJG21 study leads to an additional and unnecessary confusion rather than to a convergence of views.

## Data Availability

Any related materials regarding this study are available from the corresponding author.
